# The Global Burden of Ozone on Respiratory Mortality: No Clear Evidence for Association

**DOI:** 10.1289/ehp.1003276

**Published:** 2011-04

**Authors:** Robyn L. Prueitt, Julie E. Goodman

**Affiliations:** Gradient, Seattle, Washington, E-mail: rprueitt@gradientcorp.com; Gradient, Cambridge, Massachusetts

Anenberg et al. (2010) estimated the global burden of respiratory mortality attributable to long-term ozone exposure based on a single observational study by Jerrett et al. (2009). Because no other study has clearly demonstrated impacts of chronic ozone exposure on deaths from respiratory-related causes, we believe that reliance on the study by Jerrett et al. to establish causality and global impact is misplaced and that the conclusions of Anenberg et al. are likely unfounded.

Jerrett et al. (2009) carried out a follow-up analysis of the American Cancer Society (ACS) cohort. Other ACS studies reported no associations between long-term ozone exposure and cardiopulmonary mortality that are robust to model inclusion of co-pollutants (e.g., Krewski et al. 2000; Pope et al. 2002). In addition, other long-term studies of ozone-related respiratory or cardiopulmonary mortality did not report positive associations (Goodman 2010; Health Effects Institute 2009). Anenberg et al. (2010) suggested that long-term respiratory mortality is plausible because short-term ozone mortality has been documented, but inconsistent evidence for an association between short-term ozone exposure and respiratory mortality indicates that this relationship is not well established.

Jerrett et al. (2009) did not provide “clear” evidence of an association between long-term ozone exposure and respiratory mortality, as Anenberg et al. (2010) stated in their article. Jerrett et al. (2009) did not adequately control for potential confounding effects of particulate matter ≤ 2.5 μm in aerodynamic diameter (PM_2.5_) for several reasons. Jerrett et al. (2009) used only 2 years of data for PM_2.5_ (1999–2000) but ozone concentrations from 1977–2000. Although ozone and PM_2.5_ levels decreased considerably from 1977 to 2000, they used higher ozone levels observed in the past but only the more recent PM_2.5_ levels. Furthermore, their ozone metric focused on daily maximum hourly levels in the warm seasons, whereas they used annual average PM_2.5_ concentrations. As noted by Jerrett et al. (2009), this approach likely increased the potential to observe an association between ozone and mortality and decreased the ability to observe potential PM_2.5_ confounding of this association. In addition, confounding by other co-pollutants (e.g., sulfur dioxide), a clear issue in earlier ACS analyses (Krewski et al. 2000), was not examined. Accordingly, Jerrett et al. did not demonstrate an association between ozone and respiratory mortality that is independent of other co-pollutants.

Another aspect of the Jerrett et al. (2009) study that is inconsistent with an association between long-term ambient ozone exposure and respiratory mortality is the biologically implausible, inverse associations of ozone with cardiovascular and all-cause mortality. The magnitude of these associations is the same—although opposite in direction—as the risk estimate for respiratory mortality; thus, it is likely that associations of this magnitude are not indicative of a causal relationship.

It was inappropriate for Jerrett et al. (2009) to combine data across cities for a U.S. national risk estimate, given the known geographic heterogeneity of ozone-mortality findings (Goodman 2010). In addition, socioeconomic data (a potential confounder) was collected in 1982–1983 for the ACS study but never updated. For these reasons, the U.S. national risk estimate reported by Jerrett et al. (2009) should not be extrapolated globally.

The analysis by Anenberg et al. (2010) was based on an uncorroborated study that likely misinterpreted the findings regarding ozone effects. The utility of estimating the global burden of an effect based on a single study, for which no causal association has been established in other studies, is not apparent. Conclusions drawn from such an analysis should be interpreted with caution.
